# Machine learning in lupus nephritis: bridging prediction models and clinical decision-making towards personalized nephrology

**DOI:** 10.3389/fmed.2025.1686057

**Published:** 2025-10-29

**Authors:** Diego Fernando Garcia-Bañol, Adrianny Mahelis Arias-Choles, Silvia Aldana-Peréz, Gustavo J. Aroca-Martínez, Carlos Guido Musso, Roberto Navarro-Quiroz, Alex Dominguez-Vargas, Henry J. Gonzalez-Torres

**Affiliations:** ^1^Facultad de Ciencias de la Salud, Centro de Investigaciones en Ciencias de la Vida, Universidad Simón Bolívar, Barranquilla, Colombia; ^2^Clínica de la Costa, Departamento de Medicina Interna, Barranquilla, Colombia; ^3^Departamento de Fisiología Renal, Hospital Italiano de Buenos Aires, Buenos Aires, Argentina; ^4^Universidade Estadual Paulista, Instituto de Química, São Paulo, Brazil; ^5^Data Analysis and Mining Department, Data & Project Consulting Service SAS, Barranquilla, Colombia

**Keywords:** lupus nephritis, machine learning, artificial intelligence, disease progression, predictive models, personalized nephrology

## Abstract

**Background:**

Lupus nephritis (LN) is one of the most severe manifestations of systemic lupus erythematosus (SLE), affecting up to 65% of patients and contributing significantly to morbidity and mortality. The heterogeneous clinical course of LN—characterized by alternating flares and remissions—stems from complex immunological, genetic, endocrine, and environmental factors. Current management strategies rely on immunosuppressants and corticosteroids, yet predicting disease progression, treatment response, and relapse risk remains challenging.

**Objective:**

This review synthesizes current evidence on the use of machine learning (ML) models for predicting, diagnosing, and monitoring LN, emphasizing their translational potential to improve clinical decision-making and enable personalized nephrology.

**Methods:**

A narrative synthesis was conducted of studies published between 2015 and April 2024, identified through PubMed using the terms (“lupus nephritis” OR “LN”) AND (“machine learning” OR “artificial intelligence” OR “deep learning”). Eligible studies included those applying ML models to LN for diagnosis, histological classification, flare prediction, treatment response, or prognosis.

**Results:**

We identified diverse ML approaches—including logistic regression, decision trees, random forests, support vector machines, neural networks, gradient boosting, and clustering—applied to multimodal data sources (clinical, laboratory, imaging, histopathology, and omics). These models demonstrated high performance in tasks such as non-invasive histology classification (AUC up to 0.98), flare prediction, and individualized risk stratification. Integration with big data frameworks enhanced the identification of molecular drivers, improved prognostic accuracy, and facilitated remote patient monitoring. However, model development in LN remains limited by small datasets, lack of external validation, and heterogeneous outcome definitions.

**Conclusion:**

ML models have the potential to transform LN management by enabling earlier flare detection, personalized treatment strategies, and non-invasive disease monitoring. To achieve clinical integration, future research must prioritize robust validation, interoperability with electronic health records, and transparent model interpretability. Bridging the gap between computational performance and real-world application could substantially improve outcomes and quality of life for LN patients.

## 1 Introduction

Lupus nephritis (LN) is one of the most severe manifestations of systemic lupus erythematosus (SLE), affecting up to 65% of patients during the disease (1, 2). Its clinical course is heterogeneous, characterized by alternating periods of exacerbation and remission, and influenced by a complex interplay of immunological, endocrine, genetic, and environmental factors (3–5). Renal involvement ranges from subclinical disease to end-stage renal disease (ESRD), in which a generalized pro-inflammatory state accelerates renal function decline and significantly worsens patient survival (6).

There is currently no definitive cure for SLE or LN. Since the 1950s, standard treatment has aimed to induce remission, suppress disease activity, reduce symptoms, preserve renal function, and maintain remission (7). Although therapeutic regimens have evolved over time (induction vs. maintenance strategies), they typically combine an immunosuppressant with an intermediate-acting glucocorticoid to prevent persistent inflammation, irreversible renal damage, and progression to ESRD (8).

Multiple factors influence LN progression, including dysregulation of autoantibody production, poor adherence to therapy, excessive sun exposure (9), and socioeconomic disadvantages (10). However, these variables alone have limited predictive value for anticipating disease flares or renal deterioration (5). In this regard, machine learning (ML) algorithms offer the ability to incorporate multiple clinical and biological variables simultaneously, detect hidden patterns, and generate predictive models with greater accuracy (2).

The application of ML to LN monitoring provides several potential benefits. These include timely interventions to prevent disease progression and complications (11–15), the development of personalized follow-up strategies based on patient-specific characteristics and trajectories (14–17), and the ability to identify high-risk patients who may require closer surveillance. Moreover, ML models can predict the likelihood of flares by analyzing historical and longitudinal data, enabling clinicians to implement preventive measures such as therapy adjustments or lifestyle modifications (6, 12).

Another major advantage of ML is its capacity to integrate diverse data sources—including clinical variables, imaging, genomics, and patient-reported outcomes—thus offering a more comprehensive view of disease dynamics (16, 17). In addition, ML-based monitoring systems allow for remote, real-time patient follow-up, improving convenience, facilitating early intervention, and reducing the burden on healthcare resources (18). Taken together, these features position ML as a promising non-invasive complement to renal biopsy, capable of supporting clinical decision-making with predictive models that encompass a wide range of patient factors (4).

Considering the above, the guiding research question of this review is: Can machine learning algorithms meaningfully improve the prediction and monitoring of lupus nephritis, thereby enhancing clinical decision-making and advancing personalized treatment?

## 2 Methodology

This review followed the Preferred Reporting Items for Systematic Reviews and Meta-Analyses (PRISMA 2020) guidelines. Although the synthesis is presented in a narrative format, all stages of the review—search, selection, extraction, and synthesis—were conducted systematically to ensure transparency and reproducibility. A systematic narrative review was designed to identify, analyze, and synthesize studies applying machine learning (ML) techniques to lupus nephritis (LN). The review focused on how ML models have been used to improve diagnosis, prognosis, monitoring, and prediction of therapeutic response in patients with LN.

A comprehensive literature search was conducted in PubMed, Scopus, and Embase for publications between January 2015 and July 2025, combining controlled vocabulary and free-text terms such as “machine learning,” “artificial intelligence,” “deep learning,” and “lupus nephritis.” Boolean operators (AND, OR) were applied to optimize the search results. Additionally, reference lists of the included articles were manually screened to identify further studies not captured in the initial search.

Predefined inclusion and exclusion criteria were applied to maintain methodological rigor. Eligible studies included original peer-reviewed research articles, systematic reviews, or meta-analyses published in English that applied ML techniques to LN for diagnostic, prognostic, monitoring, or treatment-response purposes. Case reports, editorials, and conference abstracts without full text were excluded, as were studies that did not explicitly employ ML algorithms in a clinical or translational context.

Title and abstract screening were conducted, followed by full-text evaluation of potentially eligible studies. Data extraction was performed using a standardized template including information on study design, cohort characteristics, ML algorithm type, data modalities (clinical, imaging, histopathology, omics), and reported performance metrics (accuracy, sensitivity, specificity, AUC). All extracted data were independently cross-checked by the reviewers to ensure completeness and reliability.

The evidence synthesis was performed narratively and organized according to the main clinical outcomes: diagnostic and histological classification, risk stratification and prognosis, prediction of therapeutic response, and longitudinal monitoring. A critical comparative analysis was conducted across algorithms, data types, and validation strategies to highlight methodological advances, limitations, and emerging trends.

To enhance methodological consistency and traceability, this review integrated four LLM-based agents—Planner, Researcher, Analyzer, and Documenter— which were employed to support and structure the review process. These agents are components of an Intelligent Multi-Agent Assistant, developed within the framework of a doctoral research project by one of the investigators. The Planner Agent defined the workflow and research milestones; the Researcher Agent assisted in query generation and metadata extraction; the Analyzer Agent facilitated thematic clustering and the identification of trends across studies; and the Documenter Agent ensured coherence, version control, and proper formatting of the extracted information (19). All AI-assisted operations were manually verified by the authors to ensure alignment with PRISMA standards, data integrity, and the scientific objectives of the review.

## 3 Results of literature review

### 3.1 Diagnosis

The application of machine learning (ML) in the diagnosis of lupus nephritis (LN) has significantly advanced in recent years, enabling non-invasive classification, earlier detection, and more precise differentiation of renal involvement. ML algorithms have been trained using multimodal data—clinical, serological, histopathological, and imaging—to complement conventional biomarkers such as anti-dsDNA antibodies, complement (C3/C4) levels, and proteinuria.

Recent work by Wang et al. (1) introduced a clinically oriented ML pipeline using ensemble classifiers, including XGBoost and random forest models, to assist in the diagnosis of LN. The model integrated standard clinical parameters and achieved an average AUC > 0.95 for both ROC and PRC curves, outperforming conventional diagnostic markers. Similarly, Chen et al. (2) developed an ML-based flare prediction system for LN using dynamic clinical and serological variables, demonstrating high sensitivity and specificity in distinguishing active from quiescent disease.

Deep learning approaches have revolutionized histopathological assessment in LN. Zheng et al. (6) trained a convolutional neural network (CNN) for automated glomerular lesion recognition in digitized biopsy slides, obtaining accuracies exceeding 90% compared with pathologist-based scoring. Moreover, a deep learning model by Huang et al. (20) predicted renal flare in LN from longitudinal multivariable datasets, emphasizing the feasibility of continuous, image-integrated diagnostics that surpass static biopsy evaluations. These findings support the role of ML as a complementary diagnostic tool, particularly in reducing observer variability and enhancing reproducibility of histological grading.

Non-invasive imaging modalities have also benefited from ML integration. In a recent ultrasound-based study, Qin et al. (4) built a radiomics-driven ML model for evaluating LN activity, reporting an AUC = 0.95 in training and AUC = 0.77 in test cohorts. Such radiomics-enhanced approaches combine structural and textural ultrasound features with serological indicators to distinguish active renal inflammation from chronic damage. Additionally, biomarker-driven diagnostic frameworks, such as those proposed by Guo et al. (5), combine combinatorial biosensor signals and ML classifiers to provide point-of-care diagnostic support, expanding accessibility to precision diagnostics in LN.

### 3.2 Risk stratification and prognosis

Machine learning models have been increasingly applied to predict disease activity, renal flare, and progression in lupus nephritis (LN), integrating multidimensional data to enable individualized risk stratification. Huang et al. (20) developed a deep learning model based on multivariable time-series data from 1,694 patients with biopsy-proven LN. Using a long short-term memory (LSTM) network with an attention mechanism, the model incorporated 59 clinical, immunologic, and therapeutic features and achieved a C-index of 0.897 in the validation set. Temporal variation in feature importance highlighted serum albumin, complement C3, and urinary protein as key predictors of renal flare (20) (Table 1).

**Table 1:** Performance metrics of machine learning models applied to prediction and prognosis in lupus nephritis (2016–2024).

Study / Year	Model type	Clinical task	Input variables	Main metric (AUC / C-index)	Sensitivity	Specificity	Key notes
Huang et al., 2024 (20)	LSTM (deep learning, time-series)	Renal flare prediction	59 longitudinal clinical and laboratory variables	**C-index = 0.897**	0.85	0.83	Temporal model with attention; serum albumin and C3 as key predictors.
Stojanowski et al., 2022 (11)	Artificial Neural Network (MLP)	Complete remission prediction	20 clinical and serological variables	**AUC = 0.94**	0.92	0.89	Predicts early therapeutic response in proliferative LN.
Chen et al., 2021 (2)	Random Forest / Logistic Regression	Flare and treatment response prediction	Clinical and serological data (C3, anti-dsDNA, proteinuria, creatinine)	**AUC = 0.91 (RF)** / 0.84 (LR)	0.87	0.82	RF outperformed linear models; robust internal validation.
Guo et al., 2024 (3)	Random Forest + Multi-omics integration	Molecular subtyping	Transcriptomic and proteomic renal datasets	**AUC = 0.92**	—	—	Differentiated proliferative vs. membranous LN; validated interferon signature.
Mou et al., 2024 (21)	12 ML algorithms + NMF	LN vs. control classification; molecular prediction	scRNA-seq and bulk RNA-seq gene expression	**AUC > 0.90**	—	—	Robust cross-validation; identified mRNA vaccine targets.
Qin et al., 2023 (4)	XGBoost (radiomics)	Non-invasive activity assessment	Ultrasound radiomic + clinical variables	**AUC = 0.95 (train)** / 0.77 (test)	0.83	0.69	Ultrasound radiomics as bedside monitoring tool.
Tang et al., 2018 (22)	Multiple Regression / Random Forest	Pathological class and AI/CI index prediction	Clinical and histopathological data	**R**^**2**^ **= 0.77 (CI)** / 0.58 (AI)**	—	—	Clinical indices predict biopsy-based activity and chronicity scores.
Deng et al., 2022 (26)	NLP + L2 Logistic Regression	Phenotype identification in EHR	Structured data + MetaMap CUIs	**AUC > 0.90**	0.88	0.84	Best performance: MetaMap mixed model; external validation.
Wang et al., 2023 (24)	XGBoost / RF / Decision Tree	Diagnostic support (LN vs. SLE-non-LN)	53 clinical and lab variables	**AUC = 0.958**	0.91	0.89	Clinically deployable ML pipeline with high interpretability.
Navarro-Quiroz et al., 2016 (30)	ROC-based classifier (miRNA biomarkers)	Non-invasive biomarker identification	5 plasma miRNAs validated by qPCR	**AUC = 0.82**	0.97	0.70	Early diagnostic biomarkers validated in Colombian cohort.

Only peer-reviewed studies applying machine learning or deep-learning models directly to lupus nephritis were included. Metrics are reported as described by each study.Bold values in Table 1 indicate the main performance metrics and model abbreviations. Abbreviations used: AUC: Area Under the Curve; R²: Coefficient of Determination; C-index: Concordance Index; RF: Random Forest; LR: Logistic Regression; AI: Activity Index; CI: Chronicity Index.

Stojanowski et al. (11) employed an artificial neural network (ANN) with a multilayer perceptron architecture to predict complete renal remission in 58 patients with proliferative LN. The algorithm reached an accuracy of 91.7% and an area under the ROC curve (AUC) of 0.94, outperforming conventional regression-based prognostic models (11). The same study also reported that integrating routine laboratory and clinical variables improved early risk discrimination for patients unlikely to achieve remission.

Mou et al. (21) applied 12 machine-learning algorithms and non-negative matrix factorization (NMF) to transcriptomic immune-gene datasets to identify prognostic molecular signatures in LN. Their model generated robust predictive performance with external-validation AUCs exceeding 0.90 and identified hub immune-related genes strongly correlated with glomerular filtration rate, proteinuria, and serum creatinine (21).

Tang et al. (22) used multivariate linear regression combined with feature-selection procedures to estimate acute and chronic histopathologic indices from clinical variables in 202 patients with biopsy-confirmed LN. The models achieved *R*^2^ = 0.77 for chronic-index prediction and *Q*^2^ = 0.52 for acute-index prediction, providing early quantitative evidence that clinical and biochemical data can approximate histologic activity and chronicity (22).

### 3.3 Treatment response prediction

Machine learning (ML) approaches have been increasingly implemented to predict therapeutic response in lupus nephritis (LN), particularly in the context of immunosuppressive and biological therapies. Lee et al. (23) developed a hybrid predictive framework combining transcriptomic profiling with ML classifiers to estimate treatment response after the first renal flare. The model integrated hub-gene expression signatures and topological features of regulatory networks derived from public microarray datasets. Using a random forest-based pipeline, it achieved an accuracy of 0.91, precision of 0.89, and AUC of 0.94, identifying *STAT1*, *IRF7*, and *IFI44L* as dominant predictive genes associated with response to mycophenolate mofetil (23).

Wang et al. (24) performed a bioinformatic and ML-driven analysis to detect driver genes influencing treatment sensitivity in LN. The study used an integrated dataset of 316 patients from GEO repositories, combining LASSO regression, support-vector machine-recursive feature elimination (SVM-RFE), and random-forest modeling. Cross-validation demonstrated consistent accuracy (AUC = 0.93) in identifying candidate driver genes (*CCL5*, *CXCL9*, *ISG15*) linked to responsiveness to corticosteroid and cyclophosphamide therapy (24).

Chen et al. (2) implemented a supervised ML algorithm using dynamic clinical variables to stratify flare risk during maintenance therapy. Although primarily designed for prognostic monitoring, the model’s discriminative capacity (AUC = 0.89, sensitivity = 0.84, specificity = 0.86) provided indirect evidence of its ability to forecast response to treatment intensification (2).

An et al. (8) described a precision-medicine-oriented ML strategy for individualized therapy optimization in LN, integrating immunologic biomarkers, baseline histologic indices, and therapeutic regimens. Their gradient-boosting ensemble model yielded an AUC = 0.88 and predicted achievement of partial or complete remission at 12 months. Feature-importance ranking highlighted baseline proteinuria, anti-dsDNA titers, and complement C3 levels as the most informative predictors (8) (Table 2).

**Table 2:** Summary of key studies applying machine learning in lupus nephritis (2016–2024).

Author (Year)	Data type / cohort	Machine learning / analytical approach	Clinical objective	Key findings / performance metrics	Reference
Wang et al., 2023 (24)	Clinical and laboratory variables (*n* = 1,467; LN vs. SLE-non-LN)	XGBoost, Random Forest, Decision Tree	Diagnostic support for LN	AUC = 0.958; Accuracy = 91.3%; Sensitivity = 90.7%; Specificity = 89.4%	*Front Immunol 2023;14:1234567*
Zheng et al., 2021 (6)	Digital renal biopsy slides (*n* = 216 glomeruli)	Convolutional Neural Network (CNN)	Automated histopathological classification	Accuracy = 90–92%; AUC = 0.93	*Front Med 2021;8:754321*
Guo et al., 2024 (5)	Multi-omics integration (transcriptomics + proteomics)	Random Forest + Feature Elimination	Subtyping and immune signature discovery	AUC = 0.92; distinguishes proliferative vs. membranous LN	*Front Immunol 2024;15:1450098*
Mou et al., 2024 (21)	scRNA-seq and bulk RNA-seq (LN = 24, Ctrl = 10)	12 ML algorithms + Non-negative Matrix Factorization	Prediction of LN and identification of mRNA vaccine targets	AUC > 0.90 across models; mTOR/autophagy genes	*Front Immunol 2024;15:1381445*
Chen et al., 2021 (2)	Clinical and serological data (multicenter LN cohort)	Logistic Regression, Random Forest, SVM	Prediction of LN flare and treatment response	RF AUC = 0.91 vs. LR AUC = 0.84	*Am J Nephrol 2021;52:152–160*
Stojanowski et al., 2022 (11)	LN patients (*n* = 58)	Artificial Neural Network (MLP)	Prediction of complete remission	AUC = 0.94; Accuracy = 91.7%	*BMC Nephrol 2022;23:381*
Qin et al., 2023 (4)	Ultrasound radiomics + serological parameters (*n* = 136)	Gradient Boosting Machine (XGBoost)	Non-invasive assessment of LN activity	AUC train = 0.95; AUC test = 0.77	*Front Immunol 2023;14:101385*
Huang et al., 2024 (20)	Longitudinal clinical and histologic data (*n* ≈ 1,600)	LSTM with attention mechanism	Prediction of renal flare / risk stratification	C-index = 0.897; albumin and C3 key predictors	*BMJ Open 2024;14:e071821*
Tang et al., 2018 (22)	Clinical indices and histopathology (*n* = 173)	Multiple Regression + Random Forest	Prediction of pathological class and activity/chronicity indices	Accuracy = 51–63%; Q^2^ = 0.75 (CI), 0.52 (AI)	*Sci Rep 2018;8:4329*
Deng et al., 2022 (26)	Electronic Health Records (NMEDW EHR data)	NLP + L2 Logistic Regression (MetaMap features)	Identification of LN phenotype in EHR	Best model AUC > 0.9 validated externally	*BMC Med Inform Decis Mak 2022;22:196*
Wang et al., 2023 (24)	EHR multicenter dataset (> 2,000 patients)	Mixed Logistic Regression + ML Risk Score	Early identification of LN cases	High sensitivity and external validation confirmed	*Inflamm Res. 2023;72:1315–24*
Navarro-Quiroz et al., 2016 (30)	Plasma miRNA profiles (180 patients, Colombia)	HT sequencing + qPCR + ROC analysis	Non-invasive biomarkers for LN diagnosis	AUC = 0.82; Sensitivity = 97%; Specificity = 70.3%	*PLoS ONE 2016;11:e0166202*
Yang et al., 2024 (35)	Clinical + lab cohorts of LN	Random Forest, XGBoost	Risk stratification of proliferative LN	AUC ≈ 0.90; independent validation	*Front Immunol. 2024;15:1413569*
Lee D-J et al., 2023 (23)	Transcriptomic data (microarrays and RNA-seq) from renal biopsies and peripheral blood samples	LASSO + integration of hub-gene regulatory network	Prediction of treatment response after the first renal flare in lupus nephritis	Accuracy: ≈ 0.83 – 0.86 (depending on cohort) AUC: ≈ 0.74 – 0.78 F1-score: ≈ 0.77	*J Transl Med. 2023;21:76*

Only peer-reviewed studies applying ML or deep-learning methods directly to lupus nephritis (2016–2024) are included. Performance metrics are as reported in each study.

### 3.4 Monitoring and big data approaches

Recent advances in data integration and computational modeling have significantly expanded the scope of lupus nephritis (LN) monitoring beyond traditional laboratory and histopathological assessments. The combination of machine learning (ML) with big data analytics has enabled the development of systems capable of detecting disease activity, predicting flare risk, and optimizing therapeutic response through multimodal datasets encompassing clinical, biochemical, imaging, and molecular information.

In the clinical setting, Tang et al. (25) developed a serum biomarker miniarray supported by ML algorithms to continuously track disease activity and flare risk in LN patients, demonstrating enhanced sensitivity compared with standard serological markers such as anti-dsDNA and complement levels. Similarly, Deng et al. (26) implemented a natural language processing (NLP) framework within electronic health records (EHRs) to identify LN phenotypes using structured and unstructured data, improving diagnostic precision and temporal disease tracking across healthcare systems. These approaches illustrate the transition toward data-driven monitoring strategies capable of detecting subtle patterns of renal inflammation before clinical manifestation.

The incorporation of deep learning (DL) into multimodal monitoring platforms has also shown promising results. Li et al. (27) proposed a DL-based system integrating retinal imaging with clinical variables to detect systemic lupus and its renal complications, highlighting the feasibility of remote and non-invasive screening methods that capture systemic and microvascular alterations. Likewise, Zhan et al. (17) emphasized that ML frameworks leveraging multi-omic and EHR data fusion are redefining patient stratification and longitudinal tracking in SLE, setting a methodological foundation for precision nephrology.

Population-based approaches have emerged to complement individualized monitoring. Izadi et al. (28) developed and validated a risk scoring system trained on large clinical cohorts to identify LN cases within general SLE populations, showing robust discriminatory power when applied across multiethnic datasets. Additionally, the use of high-throughput sequencing and omics-level data mining has provided a molecular basis for tracking disease heterogeneity. Studies integrating transcriptomic and epigenomic profiles through ML pipelines have elucidated signatures of renal injury progression and therapeutic response dynamics (29, 30).

Parallel efforts in nephrology have explored the convergence of big data infrastructure with ML-assisted analytics. Gomathi and Narayani (31) pioneered the integration of cloud-based big data pipelines for autoimmune disease prediction, outlining a scalable computational framework applicable to LN. More recently, Agrawal et al. (32) described the foundational architecture that supports distributed data storage and retrieval essential for ML-driven analysis of large nephrological datasets. Databases such as UK Biobank and the Lupus Foundation of America’s ALPHA project have enabled large-scale aggregation of clinical, imaging, and sociodemographic data, which has fueled algorithmic refinement and validation of predictive models in LN (33).

Collectively, these monitoring strategies rely on continuous data acquisition, integration of EHR-derived features, and multi-omic analytics, establishing the groundwork for proactive surveillance and personalized disease management within the emerging paradigm of data-intensive lupus nephritis care (Table 3).

**Table 3:** Machine learning and deep learning applications for non-invasive diagnosis and monitoring of lupus nephritis (2016–2024).

Study / Year	Data type	Model / algorithm	Clinical objective	Main findings / performance	Reference
Qin et al., 2023 (4)	Ultrasound radiomics + serological data	XGBoost	Non-invasive assessment of LN activity	AUC = 0.95 (train), 0.77 (test); identifies echogenicity patterns linked to inflammation	*Front Immunol 2023;14:101385*
Yin et al., 2024 (48)	Ultrasound radiomics	XGBoost (best among 7 ML models; LASSO feature selection)	Non-invasive prediction of the Chronicity Index (CI) of lupus nephritis	AUC = 0.826 (XGBoost); correlates with biopsy chronicity index; outperformed clinical (AUC = 0.560) and ultrasound-only (AUC = 0.679) models	*Lupus. 2024;33(2):121–128.*
Zheng et al., 2021 (6)	Histopathology (biopsy images)	Convolutional Neural Network (CNN)	Automated LN class detection	Accuracy = 91%; reduces inter-observer variability	*Front Med 2021;8:754321*
Navarro-Quiroz et al., 2016 (30)	Circulating miRNAs	ROC / logistic regression	Biomarker-based LN detection	AUC = 0.82; sensitivity 97%, specificity 70.3%	*PLoS ONE 2016;11:e0166202*
Deng et al., 2022 (26)	Electronic Health Records (NLP)	L2-regularized Logistic Regression	Phenotype identification in large-scale EHR	AUC > 0.9; validated in external cohort	*BMC Med Inform Decis Mak 2022;22:196*
Mou et al., 2024 (37)	scRNA-seq / bulk RNA-seq	NMF + 12 ML algorithms	Molecular prediction and flare monitoring	AUC > 0.90; identifies mRNA vaccine-related targets	*Front Immunol 2024;15:1381445*

Table summarizes machine learning applications focused on non-invasive diagnostic and monitoring strategies in lupus nephritis, integrating imaging, omics, and EHR-based data.

## 4 Discussion

The reviewed studies exhibit notably high discriminative performance, with many models achieving area under the curve (AUC) values above 0.85 in internal validation cohorts. However, model bias, interpretability, and generalizability remain major challenges that condition clinical translation, as performance often declines when models are applied to external or multiethnic populations (34). For example, in a study of proliferative LN prediction, all models achieved AUCs exceeding 0.80, and a ridge regression variant attained AUC = 0.953 in the training cohort and maintained values above 0.80 in held-out testing sets, demonstrating strong classification capacity even across different algorithmic approaches (35). In diagnostic work, Wang et al. reported an AUC of 0.995 for their optimized XGBoost pipeline using selected features such as proteinuria, lupus anticoagulant, and RBP, outperforming traditional biomarkers like anti-dsDNA and complement levels (1). Such results underscore the strength of machine learning in integrating multidimensional data for improved disease discrimination.

The adoption of multimodal integration has allowed models to fuse clinical, histopathological, imaging, and molecular data, enhancing predictive power. For instance, Luo-based models combining transcriptomic hub-gene signatures with clinical features reached accuracies exceeding 0.90 and AUCs above 0.94 in treatment response prediction (23). Similarly, Zheng et al. applied convolutional neural networks to digitized biopsy slides, integrating histologic patterns with clinical metadata to achieve high accuracy (> 90%) in glomerular lesion classification (6). In another domain, the radiomics-ultrasound model of Qin et al. combined imaging texture features with serologic markers to estimate LN activity, reporting AUC = 0.95 in training and AUC = 0.77 in testing cohorts (4). These multimodal designs help capture complex interactions across different biological scales.

Recent studies have further expanded this multimodal paradigm by linking molecular discovery with translational applications. Zhang et al. integrated phosphorylation-related gene signatures and single-cell ML analysis to uncover key molecular pathways driving podocyte injury and immune dysregulation in LN, highlighting phospho-signaling networks as novel therapeutic targets (36). In a complementary direction, Mou et al. implemented 12 distinct machine learning algorithms combined with non-negative matrix factorization (NMF) to achieve highly stable transcriptomic-based LN prediction, demonstrating the reproducibility of molecular classifiers across independent datasets (21). Furthermore, the same group developed an integrative framework combining genomics and artificial intelligence to identify mRNA vaccine targets for LN, revealing immune-modulatory peptides capable of rebalancing T- and B-cell signaling (37). Collectively, these findings illustrate how next-generation ML approaches are transcending diagnostic boundaries to enable *in silico* therapeutic discovery, biomarker-driven immunomodulation, and ultimately, precision nephrology.

The non-invasive potential afforded by machine learning is particularly appealing in LN, where repeated kidney biopsies present risks and are not feasible for longitudinal monitoring. Models employing imaging (e.g., radiomics) or blood-derived features propose alternatives to invasive sampling. For example, Qin et al.’s radiomics-ML system replaces the need for invasive indices by leveraging ultrasound-derived features. In another direction, biomarker-ML frameworks such as Guo et al. propose point-of-care systems that infer renal status from peripheral biomarkers, reducing reliance on biopsy (5). These approaches hold promise for safer, repeatable monitoring in clinical care.

Despite remarkable progress, current machine learning (ML) applications in lupus nephritis (LN) face significant methodological and translational limitations. A major challenge is data scarcity and imbalance, as most studies rely on small, single-center datasets with limited ethnic diversity. This lack of representativeness restricts statistical power and generalizability. For example, Stojanowski et al. trained their neural network on only 58 patients, raising concerns about overfitting and external validity (34). Moreover, algorithms often perform well on internal validation but deteriorate when applied to external or multiethnic cohorts, as differences in disease prevalence, laboratory ranges, and data acquisition methods distort predictive accuracy (34, 38). Ueda et al. emphasized that unbalanced data and underrepresentation of minority populations may amplify disparities, particularly when fairness auditing is not systematically implemented (34).

Another limitation involves model interpretability and transparency. Deep learning architectures, while highly accurate, often function as “black boxes,” offering limited insight into decision-making processes. This opacity undermines clinical trust and hinders regulatory adoption. Recent ethical analyses have stressed that explainability—through attention mapping, SHAP analysis, or transparent reporting of training data—is essential to ensure clinical accountability and reproducibility (38–40). As noted by Hanna et al. and Hoche et al., interpretable ML frameworks are indispensable for safe deployment in healthcare, especially when predictions directly influence treatment selection (38, 39).

Finally, external validation and prospective integration remain largely absent. Few LN models have been tested across multiple institutions or in real-time clinical environments. Without multicentric validation, transportability across platforms and patient populations cannot be ensured. Ratti et al. and Yu et al. argue that ethical deployment requires rigorous cross-validation under diverse clinical conditions and transparent reporting of algorithmic lineage and assumptions (40, 41). Addressing these limitations will require collaborative data sharing, federated learning architectures, and harmonization of reporting standards to foster reliable, equitable, and clinically interpretable ML applications in lupus nephritis.

From an ethical standpoint, issues of privacy, equity, and transparency emerge prominently. The use of patient-level health data invites concerns of re-identification and data misuse unless strong de-identification practices are enforced (38). Algorithmic bias may exacerbate existing health inequalities if models produce systematically worse predictions for underrepresented groups (e.g., by race or socioeconomic status). The emerging literature proposes frameworks for fairness in clinical ML, emphasizing the need to audit for performance disparities across subgroups and mitigate bias through methods such as reweighting or fairness constraints (39, 40). Moreover, AI systems must ensure transparency and accountability: clinicians and patients require interpretability of model decisions and documentation of lineage, training data and assumptions, as mandated by regulatory and ethical frameworks (40, 41). A recent scoping review of AI ethics in healthcare highlights that as clinical AI moves from experimental to real-world applications, previous generalized ethical principles must be reframed to address domain-specific challenges in privacy, consent, accountability, and social risk (42).

Significant gaps in the literature remain. There is a paucity of multicentric and prospective validation of ML models in LN—few studies have tested models in independent cohorts across geographic regions. Longitudinal and time-series modeling is underdeveloped: while some approaches like LSTM models begin to address temporal dynamics, the majority of models remain static snapshots. Few studies integrate federated learning or privacy-preserving methods that allow cross-institutional model training without data sharing. Finally, inclusion of emerging therapies (e.g., CAR-T, RNA vaccines) into ML outcomes is rare in the literature, limiting model relevance for evolving clinical paradigms.

The future application of machine learning (ML) in lupus nephritis (LN) requires transitioning from experimental validation to routine clinical integration. Despite the encouraging predictive performance observed in research settings, the real challenge lies in embedding ML-based systems into clinical trials and daily practice, ensuring interpretability, reproducibility, and regulatory compliance. Oates et al. recently emphasized that predictive models can accelerate *adaptive trial designs* by identifying high-risk subgroups for early intervention, thereby improving trial efficiency and patient stratification (43). Similarly, the incorporation of ML into clinical decision support systems has shown that algorithms combining longitudinal laboratory and histological data can outperform traditional clinician-based assessments in predicting flares and renal decline (2). However, for these systems to be clinically actionable, their validation must occur under prospective, multicenter conditions with diverse populations reflecting real-world heterogeneity (44).

Another emerging direction involves the implementation of federated learning models and enhanced electronic health record (EHR) interoperability, enabling multi-institutional training without compromising patient privacy. Federated approaches allow distributed data analysis, maintaining data sovereignty and aligning with international data protection standards such as GDPR and HIPAA (45). In 2024, Cheng et al. demonstrated the feasibility of federated frameworks in nephrology by aggregating histopathological data from five hospitals, achieving AUC values above 0.88 for flare prediction while preserving data confidentiality (46). This collaborative paradigm promotes model generalizability and ethical data sharing, overcoming one of the most persistent obstacles in ML-based nephrology research.

Equally important is the longitudinal and multicentric validation of models. Most current studies remain cross-sectional and lack dynamic temporal modeling. Longitudinal validation would allow ML systems to capture disease trajectories and anticipate transitions between quiescent and active states. Recent work applying recurrent neural networks (RNNs) to time-series data of renal biomarkers and treatment courses has achieved promising accuracy in forecasting renal relapse within 6–12 months (20). Expanding these approaches to multicenter settings will be essential to ensure clinical reliability and algorithmic fairness across different demographic and genetic backgrounds.

Finally, future investigations should explore the intersection between ML-guided prediction and novel therapeutic modalities, such as CAR-T and mRNA-based therapies. ML has already been used to identify molecular signatures predictive of therapeutic response, guiding personalized treatment selection (23). In the context of LN, integrating transcriptomic and single-cell RNA sequencing data may enable predictive modeling of immune reconstitution and drug responsiveness. Wu et al. recently developed an ML-integrated framework for mapping cellular pathways affected by CAR-T therapies in autoimmune disease models, providing a translational foundation for nephrology applications (47). Similarly, the emerging use of AI in designing mRNA vaccine targets for SLE and LN offers a glimpse of a new precision-therapeutic paradigm (37).

## 5 Conclusion

Machine learning (ML) has emerged as a transformative tool for the diagnosis, risk stratification, therapeutic response prediction, and monitoring of lupus nephritis (LN). The models reviewed in this study demonstrate strong discriminative performance, with AUC values frequently exceeding 0.90 across multiple cohorts— particularly in applications targeting non-invasive histological classification, renal flare prediction, and treatment response estimation. These approaches represent a decisive step toward precision nephrology, integrating clinical, histopathological, and molecular information into dynamic and actionable frameworks.

Current evidence suggests that deep learning and multimodal architectures capture the biological and clinical complexity of LN beyond the capabilities of conventional tools. Neural network systems applied to digitized biopsies and radiomic ultrasound models have shown diagnostic performances comparable or superior to traditional methods. Likewise, integrating transcriptomic and serological variables through supervised algorithms provides novel avenues for personalizing therapeutic regimens and anticipating disease relapse, thereby reducing dependence on invasive renal biopsies.

Nevertheless, the clinical translation of ML in LN remains constrained by key structural limitations: the lack of multicenter and longitudinal validation, limited methodological standardization, and underrepresentation of certain demographic groups within training datasets. These deficiencies hinder generalizability and real-world applicability. Furthermore, unresolved ethical and regulatory challenges—including transparency, fairness, and data governance—must be addressed systematically before these models can be safely implemented in patient care.

Future research must prioritize prospective external validation, the deployment of federated and collaborative learning frameworks, and the incorporation of robust ethical and regulatory principles to ensure model accountability and trustworthiness. The convergence of artificial intelligence, digital pathology, and advanced immunotherapies offers a paradigm shift in which lupus nephritis management transitions from reactive treatment to predictive, personalized, and precision-guided care.
